# A Gap or Lacuna in the World Anti-Doping Code? Remarks on the CAS Interpretation in IOC, WADA, and ISU v. RUSADA, Kamila Valieva and Russian Olympic Committee (CAS OG 22-08, CAS OG 22-09, and CAS OG 22-10)

**DOI:** 10.3389/fspor.2022.946608

**Published:** 2022-07-05

**Authors:** David Pavot

**Affiliations:** Professor of Law, Research Chair on Anti-Doping in Sport, Department of Marketing, Business School, University of Sherbrooke, QC, Canada

**Keywords:** Word Anti-Doping Code (WADC), Court of Arbitration for Sport (CAS), lacuna, protected person, interpretation

## Abstract

This article focuses on the award of 14 February 2022, rendered by the CAS *ad hoc* chamber in the appeal filed by IOC, ISU and WADA against the decision of the RUSADA Disciplinary Committee lifting the provisional suspension of Kamila Valieva. Kamila Valieva is one of the world's best 15-year-old figure skaters. Prior to the Beijing Olympic Games, she tested positive for a substance prohibited in and out of competition under the WADC and was provisionally suspended. Her suspension was lifted by the RUSADA Disciplinary Committee, and the case was brought before the CAS. Because of her young age, Valieva is a minor and therefore a protected person according to the WADC. The CAS concluded that the Code, by setting up a system of sanctions for these protected persons but not providing for any temporary sanctions, contained a lacuna that it had to fill. However, the identification of the gap and the interpretation of the rules by CAS is questionable in this award.

## Introduction

On 14 February 2022, the Court of Arbitration for Sport (CAS) rendered an award in the mediatized case of the new Russian figure skating prodigy, Kamila Valieva, who is 15 years old and therefore considered a protected person under the World Anti-Doping Code (WADC). In its award, the CAS decided to reject the appeal against the decision of the Russian Anti-Doping Agency (RUSADA) Disciplinary Committee lifting the Athlete's suspension following a positive doping test. One of the central elements of the decision was that the legal regime for protected persons was deficient because it does not contain a specific provision for provisional suspensions. The argument of this article is that the interpretation of the notion of a gap or lacuna in the WADC by the Panel is problematic as it does not correspond to the definition, nor to the previous CAS jurisprudence the panel intended to follow. First, we will recall the key elements of the Valieva case. In a second step, we will present the legal regime of gaps and discuss them in the context of the WADC. In a third step, we will question the application of the legal regime of gaps that the panel indicates using in Valieva by arguing that the interpretation is not consistent.

## The Valieva Case in a Nutshell

The Kamila Valieva saga, which ended with a disappointing performance in the individual figure skating event, was one of the media whirlwinds of the 2022 Beijing Winter Olympic Games. To understand the case, it is necessary to go back in time.

On 25 December 2021, Kamila Valieva brilliantly won the women's individual competition during the Russian figure-skating championships and was designated for a post-event doping test. Due to the suspension of the Moscow Anti-Doping laboratory, the sample was sent for analysis to the Karolinska Institute in Stockholm, Sweden. On 7 February 2022, she was a member of the team who won the Olympic title in the figure-skating team event. On 8 February, she was notified of the result of the test conducted on 25 December 2021, which was positive for Trimetazidine, a substance prohibited in and out of competition under the WADC and was given a provisional suspension by RUSADA. It was then learned that the International Olympic Committee (IOC) would not hold a medal ceremony due to legal issues.

The athlete then decided to challenge the suspension before the RUSADA Disciplinary Committee. The argument developed by the athlete and his counsel, and retained by the Committee, revolved around inadvertent doping. In this sense, the athlete's grandfather claimed that he was taking Trimetazidine to treat himself and that contamination could have occurred during a glass exchange (CAS OG 22/08, 23). This interpretation was accepted by the Disciplinary Committee, which lifted his provisional suspension on 9 February (CAS OG 22/08, 33–36).

On 11 February, the World Anti-Doping Agency (WADA), the IOC and the International Skating Federation (ISU) appealed to the Court of Arbitration for Sport (CAS) to overturn the decision of the Disciplinary Committee against RUSADA. In an award of 14 February, which was widely commented on in the media and in the world of sport, the CAS rejected the Appeal.

From a procedural point of view, during the Olympic Games, the CAS creates an *ad hoc* chamber with arbitrators who are dispatched during the entire duration of the Games to decide cases with all due speed. It is therefore not necessarily arbitrators from the antidoping division who deal with cases concerning possible antidoping rule violations (ADRV's). Notwithstanding this, no criticism of the competence of the arbitrators on the panel was made by the parties. At most, Kamila Valieva's counsel questioned the appointment of Jeffrey Benz, a former senior legal advisor to the United States Anti-Doping Agency (USADA), but the matter was quickly resolved (CAS OG 22/08, 54).

The panel was essentially asked to reverse the decision of the RUSADA Disciplinary Committee (CAS OG 22/08, 70–76). In particular, the appellants wanted the arbitrators to review the decision because they considered that Ms Valieva's status as a Protected Athlete under the WADC could not, on its own, justify such a lifting of her provisional suspension and a lowering of the burden of proof regarding her allegations of inadvertent doping. It should be noted that Protected Athlete Status is an innovation of the 2021 WADC. It establishes a system of reduced sanctions for minors or persons even if they have reached the age of majority and lack legal capacity under their national law (World Anti-Doping Code 202, 2021, annex 1 and art. 10.6.1.3). In addition, WADA relied on the fact that the substance in question was prohibited both in and out of competition, which increased the burden of proof on the athlete to lift a provisional measure (CAS OG 22/08, 56–96).

CAS rejected the appeal and upheld the decision of the RUSADA Disciplinary Committee, thus allowing the athlete to continue competing. The essence of the panel's reasoning is that the WADC, while containing provisions to give preferential treatment to protected persons with respect to sanctions, does not provide any information regarding provisional suspensions of protected persons. According to the arbitrators, this absence constitutes a lacuna in the WADC (CAS OG 22/08, 200) that could cause irreparable harm to the athlete in this case (CAS OG 22/08, 205).

As soon as the decision was published, the appellants were highly critical of the CAS award. The IOC considered this to be an “inconclusive situation” and decided that the medal ceremony in the team figure skating event would only be held once the case of an ADRV had been decided on its merits and that, should Valieva win an individual medal, the same would apply (International Olympic Committee, 2022). For its part, WADA expressed its disappointment and lack of understanding by noting that “it appears that the CAS panel decided not to apply the terms of the WADC, which does not allow for specific exceptions to be made in relation to mandatory provisional suspensions for “protected persons', including minors” (World Anti-Doping Agency, 2022). The antidoping community was also shaken (Pavot, 2021), and some did not hesitate to link this case to the state-run doping system in Russia.

## The Legal Regime of Lacuna Based on WADC

### The Question of Lacuna in law

Historically, the question of the legal lacuna has arisen in the context of the civil law legal systems based on written codes (Irti, 2014, p. 157–179). Therefore, it not surprising it has been raised in the context of the WADC. From a theoretical, even philosophical point of view, this is also an important debate since it refers to the completeness (Troper, 2011, p. 19–29) and coherence of legal systems (Broekman, 1985, p. 218). The debate is more important because, despite the successive revisions of the Code, there is no permanent drafting body for it, just as there is no permanent and unique jurisdiction in the matter, even though CAS is considered the “supreme court of world sport” (Swiss Federal Tribunal, 2003, 4P.267–270/2002, 3.3.3).

The definition of the legal gap has been discussed in the literature and today it is widely accepted that “there is a legal lacuna (or lacuna in law) when there is no rule in the legal system that the judge can use to resolve a given case” (Foriers, 1967, p. 58). Thus, “lacuna imply that [none of the existing rules] provides a solution for the case in question that one seeks to resolve in accordance with the law in force” (Siorat, 1959, p. 256). These lacunas are to be distinguished from “false lacuna” which exist in the presence of a “rule appearing in the legal system to resolve a given case which does not appear to be appropriate, satisfactory or just”; they are considered “false since there is necessarily a rule but that this rule is going to be set aside by the person who has the task of judging” (Siorat, 1959, p. 257).

### Lacuna and WADC: Lessons From the Puerta Case

According to dictionaries, a lacuna is defined as a deficiency, an oversight, what is missing to complete something (Collins^1^; Merriam-Webster).^2^ On its side, the CAS has used the lacuna to fill in what it considered to be gaps in the Code, but on very rare occasions. Indeed, the conditions for using this technique were defined by the Puerta award in which the Panel clearly outlines the procedure to follow while specifying that “There may be other circumstances in which a tribunal would be tempted to find a gap or lacuna in the WADC, but the Panel has found it difficult to imagine that such cases will frequently arise” (CAS 2006/A/1025, p. 95). Subsequently, the Panel sets out very strict cumulative conditions: (1) the gap in the Code must be related to the specific case and (2) can only occur in very exceptional circumstances and should never occur again. Once identified, (3) the gap must be filled using general principles and (4) must not weaken either the Code or WADA.

In Valieva, The CAS makes it clear in its reasoning that it falls within the conditions listed in Puerta by explicitly quoting the sentence. “As a result, it appears to the Panel there is a *lacuna*, or a gap, in the Russian ADR, and indeed the WADC 2021.” When CAS panels find a *lacuna* in the WADC, this has been the basis for a CAS panel to find a gap filling construct that would ameliorate an overly harsh or inconsistent outcome: “*[W]hen there is a gap or lacuna in the WADC, that gap or lacuna must be filled by the Panel . . . applying the overarching principle of justice and proportionality on which all systems of law, and the* [WADC] *itself, is based*.” (CAS 2006/A/1025; CAS OG 22/08, 200). However, the panel very quickly disassociates itself from them both in the process of identifying the gap and in filling it. This is unfortunate because the conditions listed in Puerta allowed the approach to be legitimized by corresponding to the legal definition of the lacuna, while at the same time framing the use of a potentially dangerous technique. Articulating its reasoning on a lacuna in the Code in relation to the absence of a specific provision for provisional suspensions for protected persons, the CAS, should have followed the approach developed in the Puerta award. It is true that CAS panels are not bound by the rule of precedent. However, it has been repeatedly shown that there is a very strong tendency to respect the decisions of previous panels (Bersagel, 2012). More generally, although arbitral tribunals sometimes use the excuse of not being bound by the rule of precedent to depart from previous awards, they tend to respect it to develop a coherent and predictable jurisprudence for litigants (Kauffman-Kohler, 2007). CAS cannot be an exception if it wants to remain the World Court of Sport (Lindolhm, 2021), especially when it acts in matters of disciplinary sanctions. Moreover, in Valieva, the arbitrators make it clear that they intend to follow the reasoning developed by their predecessors in Puerta (CAS OG 22/08, 200). Therefore, they could not claim to be following a precedent to deviate from it by erring in their arguments.it. In addition, if CAS wants to be a truly credible World Court of Sport (Lindolhm, 2021, p. 1–5) there must be a minimum of consistency and predictability in its jurisprudence, even more so when the previous decisions are well-founded, as is the case here.

## The Questionable Interpretation of the Gap or Lacuna Concept in the Valieva Award

### The Absence of a Specific Provision on Provisional Suspensions of Protected Persons: A False Lacuna

In Valieva, the gap is characterized, according to the arbitrators, simply by the fact that “exempting older athletes from mandatory Provisional Suspensions in most instances in which they might ultimately be able to establish basis for a short sanction or reprimand but not exempting younger, legally incapable, and immature Protected Persons who might be entitled to a short sanction or reprimand appears clearly to be an unintended gap in the Code” (CAS OG 22/08, 196). The panel goes on to state: “evidence was presented at the hearing that the WADC (2021) drafting committee had apparently not considered the issue of Protected Persons in the context of Provisional Suspensions and whether the standards should vary in the case of Provisional Suspensions involving Protected Persons” (CAS OG 22/08, 197).

In essence, the arbitrators rely on the argument developed by the Russian Olympic Committee, which questioned the independent members of the Code Drafting Committee, Ulrich Hass and Richard Young, by email, asking them: “whether the absence of reference to “Protected Persons” in the context of Section 7.4 of the World Anti-Doping Code has been done on purpose, or whether it could be seen as an oversight or a lacuna” (CAS OG 22/08, 139). Only one of them replied: ”Prof. Haas answered that, even though he is not allowed as a CAS Arbitrator to provide an opinion as to the interpretation of this rule, he could testify that there had been no discussion in the context of the 2021 Code revision with respect to the specific issue to coordinate the provisions on ineligibility of Protected Persons with the provision on Provisional Suspension (CAS OG 22/08, 139).

The speed and simplicity of the arbitrators' conclusion is surprising. Indeed, they never note the absence of a rule on provisional suspensions of protected persons, which exists in the field of sanctions. At most, they indicate that the application of the same regime for athletes of legal age and for protected persons regarding provisional suspensions could pose a problem of coherence since the provisional sanction could cover the supposed period of the suspension. For protected persons, the sanctions imposed will generally be reduced by half compared to the sanctions imposed for unprotected athletes. Moreover, the general provision provides that the duration of the provisional sanction is deducted from the sanction finally imposed. Therefore, this is not a lacuna characterized by the absence of rules but rather a false lacuna according to the definition presented above.

### No Mention of the Exceptional Character of the So-Called Lacuna

In Puerta, the arbitrators clearly identified the very exceptional nature of the gap and the need to use the term sparingly. Indeed, even in legal systems where arbitrators or judges have used this technique, they have always done so with great caution so as not to risk the criticism of government of the courts. A lacuna identified in an expeditious manner would amount to a substitution of the legislator and an overstepping of the judge's powers. However, in the Valieva case, it does not appear that the arbitrators used the same caution as their predecessors to justify the exceptional nature of their reasoning.

First, the Panel, immediately after identifying the existence of a gap, felt the need to justify itself by explaining that “This is an exercise in interpretation, not in rewriting rules or making policies that are better made by sporting bodies exercising proper governance. The Panel wishes to emphasize that it does not see itself as a policymaker or rulemaker, but it is properly called upon, as are courts around the world, to interpret rules and how they work” (CAS OG 22/08 201). Such an *obiter dictum* is, at first glance, surprising and seems to reflect a certain unease on the part of arbitrators, who take care to recall their mission. Indeed, this is the classic function of the dispute settlement bodies and it is constant that the panels recall this role as it has been done in the past: “The role of this Panel is as adjudicator, not legislator” (CAS 2008/A/1461, 1.3.2). Furthermore, Panels must ensure that their interpretative method is consistent with CAS precedents, and in this case, that they define and identify the gap in accordance with the *Puerta* precedent. The Panel, as explained, identifies a false gap. Therefore, the consistent interpretation ultimately leads the panel to rewrite the Code in the opposite way to what they claim by applying a new rule, not provided for in the Code, according to which provisional suspensions are optional in the case at hand (CAS OG 22/08, 201).

Secondly, the panel goes to great lengths to show that the duration of the provisional suspension would cause irreparable harm to the athlete. This is not necessarily wrong given the timing of the notification of the test and the difficulty of mounting a robust defense in such a short period of time to challenge an adverse finding. It is more surprising that the tribunal immediately concludes that the regime to be applied to protected persons is that of Article 7.4.2 of the Code dealing with optional provisional suspensions which reads as follows “a Signatory may adopt rules applicable to any event under its jurisdiction or any team selection process for which it is responsible, or where the Signatory is the relevant International Federation or has results management authority over the alleged ADRV, to impose Provisional Suspensions for ADRV other than those covered in Article 7. 4.1 prior to the analysis of the Athlete's B sample or the final hearing under Article 8” (World Anti-Doping Code 202, 2021, Article 7.4.2). However, there is a significant difference between the mandatory/automatic suspension regime, which should have applied in Valieva's case because of the type of product detected, and the optional suspension regime, which does not cover Valieva's case at all, but which the arbitrators substituted for the former based on the risk of irreparable harm, without being permitted to do so by the Code.

In this case, the arbitrators' argumentative diversions via the lacuna leads them to a dead end in their reasoning, even though their questioning of the balance of the regime (CAS OG 22/08, 205–217) imposed on protected athletes is not unfounded. The very questionable identification of a supposed gap in the Code gives the impression of a smokescreen to conceal a proven objective of balance between the sanctions applicable to protected athletes and the provisional suspensions that they incur. Moreover, the panel rightly questions the risk of irreparable harm potentially caused to the athlete (CAS OG 22/08, 205). However, there is no need to resort to the lacuna here, as opposed to a more substantiated argument. Subsequently, the arbitrators also justify their decision because of “the length of time it took for the laboratory to submit its report of an AAF involving the Athlete, the timing of that relative to the conduct of the Women's Single Skating event at the OWG 2022” (CAS OG 22/08, 206) and to conclude after a brief argument that “the likelihood of irreparable harm is present here” (CAS OG 22/08, 211). The issue of irreparable harm brought by the Panel was not interesting because it is an important point of support for provisional suspensions. However, the arbitrators' conclusion on this point is too hasty and they immediately switch to the gap. Yet, an interesting discussion on this point would have deserved to be developed, especially because of the legal regime that follows. The task of convincing the panel would have been difficult here as well.

The arbitrators then continue their reasoning in search of a hypothetical balance between the obligations of the antidoping institutions and those of the athletes due to the delay in notifying Ms Valieva of the positive test. From a temporal point of view, the arbitrators rightly consider that the result does not comply with the 20-day time limit since the institutions took almost 40 days to inform the athlete of the positive result of a doping control (CAS OG 22/08, 211). Indeed, the International Standard for Laboratories, in its 2021 version, states that “Reporting of ‘A' Sample results should occur in ADAMS within twenty (20) days” (International Standard—Laboratories, 2021, p. 105). In this case, the delay is attributable to a COVID outbreak in the laboratory responsible for the analyses (CAS OG 22/08, 14 and 98). In its submissions, WADA explains that the delay in analyzing the sample was not exceptional (CAS OG 22/08, 211) and indicates that the Standard only sets out a recommendation on time limits. The arbitrators were not convinced by this explanation and developed an argument comparing the nature of the extremely restrictive obligations on athletes to those on laboratories, which are only recommendations (CAS OG 22/08, 212). They consider that there is an unacceptable imbalance. However, the role of arbitrators is not to weigh up the obligations weighing on one or the other but to ensure that the rules are applied, if necessary, by interpreting them. In this case, the rule for the laboratory was clear in the Standard as the “should” text is clearly indicative. In law, the general method of interpretation is to use, as a first draft, the ordinary meaning of words according to a practice widely established by CAS arbitral panels (CAS/2013/A/3365, 137–144). In the wording of the Standard, everything is clear and there was no justification for the panel to conclude in this way. More broadly, the panel's sophism pitting laboratories against athletes opens a dangerous can of worms in terms of the soundness of the reasoning, which undermines the credibility of the decision. If such arguments are repeated in the future, the entire system of antidoping in sport could be constantly called into question: indeed, it will always be possible to identify an imbalance because the obligations on the persons covered by the Code are very restrictive, whereas those on laboratories and Anti-Doping organizations are less so.

In all cases, it appears that the panel's rationale fails to mention the exceptionality criterion in its reasoning, and if it wished to apply it, it did so in a haphazard and confusing manner. Yet, given the factual elements such as the delay in notification, the period of the Olympic Games, the short time to defend, this was a potentially convincing argument which the panel missed, leaving its reader to reach its own conclusion. In the wake of its omission of the qualification of exceptionality, the panel continues in the process of filling the gap.

### The Lack of Recourse to General Principles in Filling the Alleged Gap

In the presence of a lacuna, it is customary in the various legal systems to resort to general principles to fill a legal gap (Siorat, 1959, p. 256). In this respect, the law applicable before the *ad hoc* divisions of the CAS is governed by Article 17 of the Arbitration Rules for the Olympic Games, which explicitly allows recourse to these principles: “the Panel shall rule by virtue of the Olympic Charter, the applicable regulations, the general principles of law and the rules of law whose application it considers appropriate” (CAS, Arbitration Rules applicable to the CAS *ad hoc* division for the Olympic Games). Furthermore, it has long been accepted that “any action taken against a competitor in relation to doping must respect the principles of international and national law, as well as the laws governing the protection of personality and human rights” (TAS 93/109, 0000, 5.). Finally, regarding the gap, the panel explicitly refers to the methodology developed by the Puerta case: “applying the overarching principle of justice and proportionality on which all systems of law, and the [WADC] itself, is based” (CAS 2006/A/1025, 1). However, once the gap is identified, the panel never explicitly mentions any general principle to fill it.

#### The Impossible Use of the Principle n Dutio Contra Proferentem

The Russian Olympic Committee had twice, following its argument about a gap in the Code's provisions, suggested the application of the principle (CAS OG 22/08, 134 et 142). Known in contract law as “interpretation against the drafter”, this principle provides that in case of ambiguity in a clause, the meaning retained is the one that goes against the interests of the party who imposed it. It has already been put forward before CAS by parties challenging sanctions for doping (CAS/2016/4839), has been retained in cases of player transfers (CAS 2018/A/5950) and the Swiss Federal Tribunal has also recognized its admissibility in cases where the applicable rules are ambiguous (Swiss Federal Tribunal, 2014, 4A_90/2014). However, ambiguity is not a shortcoming in the sense of the definition of the latter. Therefore, this principle could not be applied in the present case and if the arbitrators had retained it, they would certainly have been open to further criticism.

#### Are There Other Principles That Could Have Been Considered by the Panel?

The search for a principle other than the one put forward by one of the parties to the dispute could have been conducted by the Panel because it was not limited by the arguments raised before it to find the solution to the case (CAS 2005/A/951). For example, the Panel could have attempted to discover a general principle of protection of protected persons arising from the Code and to consider that the Code was a general principle of consistency. The arbitrators could also have attempted to develop an argument based on the regime's inconsistency with the principle of proportionality of sanctions, which is a widely accepted principle in sport (CAS 98/200). To do so, the arbitrators could have relied on the reasoning they develop in the award on the duration of the provisional sanction, which could exceed the final sanction. Based on the CAS case law, this principle could have been applied, or even seriously discussed, in Valieva, as the Knauss award suggests by explicitly targeting young athletes: “the purpose of introducing the WADC was to harmonize at the time a plethora of doping sanctions to the greatest extent possible and to un-couple them from both the athlete's personal circumstances (amateur or professional, old or young athlete, etc. . ..) as well as from circumstances relating to the specific type of sport (individual sport or team sport, etc.” (CAS/2005/A/847). The arbitrators, however, did not choose to go down this road, which would also surely have given rise to debate.

#### The Difficult Path of Recourse to Fundamental Rights

Another option would have been to shift to the field of general principles deriving from fundamental rights. While this avenue is possible (TAS 93/109), it remains tenuous according to practice (Maisonneuve, 2017), although the trend toward taking human rights into account is increasingly prevalent in sports justice. However, this would have been an extremely slippery slope for the panel. Indeed, in the context of the draft 2021 Code, questions relating to the status of protected persons had been submitted to Judge Costa, who considered, in a legal opinion, that “the concept of protected person is in line with the standards” (Costa, 2019). At most, he asked for clarification of the categories referred to in the Statute which have been made in the Code. In his review, Judge Costa found, inter alia, that protected person status was “either an aggravating factor in a violation committed by an athlete or other person if it involves or harms a protected person (passive protection); or a mitigating factor in a violation if, on the contrary, the protected person is the perpetrator of the violation (active protection). It does not seem to be a problem in either case' (Costa, 2019, p. 17). In terms of definitions, the expert considers that “as regards the definitions of protected persons, the draft 2021 Code has also been amended since Appendix I now specifies that the age below which a person is protected is 16 years, and 18 years if he or she has never participated in an international competition open to adults. This is satisfactory” (Costa, 2019, p. 36). Furthermore, in terms of sanctions, it states that “if the protected person can establish that he or she has committed an insignificant fault or negligence, he or she may be subject to a sanction ranging from a minimum of a reprimand, without suspension, to a maximum of two years' suspension.” Overall, Judge Costa's analysis, based on an examination of the rules and case law on fundamental rights, gives a positive response to the provisions of the Code, without ever identifying any shortcomings about provisional suspensions for protected persons.

Consequently, even if the panel had correctly identified the shortcoming, recourse to the general principles would have required a drafting effort that it unfortunately did not make. On the contrary, the panel, in an *obiter dictum*, takes advantage of the opportunity to scratch the Code and WADA, deviating, once again, from the path traced by its predecessors.

## An Award Weakening WADA and the Code

If a lacuna is found, it must not be intended to “weaken WADA or WADC” (CAS 2006/A/102, 101). However, both in the principle of the decision to question the very principle of provisional suspension for protected athletes and in the questioning of the antidoping ecosystem in the award, the conclusions of the arbitrators contradict the statement.

Indeed, provisional suspensions pursue the objective of protecting “the integrity of competition, the Anti-Doping Organization must also protect participants from an opponent who has potentially cheated. There is no way to compensate the non-tainted athlete whose place has been taken by the drug cheat [...]” (Lewis and Taylor, 2021, p. 725). Thus, one court has already stated that “in coming to this conclusion the Panel has also considered the interests of the Athlete in not being able to compete and possibly obtain a medal as well as of the other athletes who would be deprived of their opportunity to be awarded medals at the Rio Olympic Games should the Athlete successfully medal and is later determined to have committed an ADRV. The Panel has further considered the interests of sports in general and the IAAF, noting the importance of protecting the image of sport from being tarnished by the participation of athletes in competitions who are facing proceedings against them for the use of prohibited substances” (CAS OG 16/23, 7.11). In doping matters, CAS has already considered that “the suspension of the effects of the contested measure must be ordered sparingly” (TAS 2005/A/958).

In the Valieva case, it does not appear that the arbitrators took the necessary precautions to protect the rights of other athletes and the integrity of the system. On the contrary, they allowed an athlete who tested positive to compete considering the recorded testimony from her grandfather attesting only to the fact that he was taking medication containing a prohibited substance. At no time during the RUSADA proceedings or before the CAS was this witness able to be cross-examined (CAS OG 22/08, 23, 31, 70, 78 and 93) and the fact that a relative of the athlete was taking medication cannot reasonably lead to an automatic admission of contamination.

Furthermore, the arbitrators, in their decision, do not hesitate to scratch the Anti-Doping system by explaining “when athletes are held to a high standard in meeting their Anti-Doping obligations and at the same time, the Anti-Doping authorities are subject to mere recommendations on time deadlines that are designed to protect athletes from late- or inconveniently arising claims. The flexibility of the recommendations and guidelines applicable to WADA-accredited labs contrasts with the stringency of the rules on Provisional Suspensions” (CAS OG 22/08, 211). From an argumentative point of view, such a sophism looks like a direct and voluntary undermining of the institutions and its utility is more than questionable. Furthermore, the arbitrators go on to clearly blame the Anti-Doping authorities: “this has been the result of the relevant Anti-Doping bodies to ensure timely analysis of pre-Games samples and failing to ensure that pending cases are resolved before the Olympic Winter Games commence” (CAS OG 22/08, 212) even speaking at one point of “failure of the Anti-Doping authorities” (CAS OG 22/98, 201). Overall, these passages constitute an unnecessary attack on the system and undermine the credibility of the decision, while at the same time falling outside the lines drawn in the Puerta sentence.

## Conclusion

At the end of our analysis, the arbitral tribunal's argument is flawed. It does not correspond to what is announced, nor to CAS precedents, nor to the definitions of what is a legal gap. In many respects, it seems that the award was drafted based on a solution constructed before the legal argument, which had to be identified later. It is also surprising that the court essentially adopted the conclusions that had been presented by Bill Bock, USADA former General Counsel, even before the panel had rendered its decision. The least that could have been done would have been to refer to the text that appeared on the lawyer's law firm blog (Bock, 2022)—although it has strangely disappeared today.

At the end of the 2020 Tokyo Games, the WADA Independent Observer Report regretted an “insufficient level of anti-doping knowledge by some members of the CAS ADD, specifically their understanding of some provisions of the Code, the International Standard for Results Management, as well as CAS jurisprudence on strict liability, burden of proof, and responsibility of that burden” (World Anti-Doping Agency, 2020, p. 36) even though some of the arbitrators presents were on the CAS Anti-Doping list. Time will tell if such a comment will be present in the report on the Beijing games.

## Data Availability Statement

The original contributions presented in the study are included in the article/supplementary material, further inquiries can be directed to the corresponding author/s.

## Author Contributions

The author confirms being the sole contributor of this work and has approved it for publication.

## Conflict of Interest

The author declares that the research was conducted in the absence of any commercial or financial relationships that could be construed as a potential conflict of interest.

## Publisher's Note

All claims expressed in this article are solely those of the authors and do not necessarily represent those of their affiliated organizations, or those of the publisher, the editors and the reviewers. Any product that may be evaluated in this article, or claim that may be made by its manufacturer, is not guaranteed or endorsed by the publisher.

## Footnotes

^1^Collins Dictionary, “Lacuna”. https://www.collinsdictionary.com/dictionary/english/lacuna.

^2^Merriam-Webster Dictionnary, “Lacuna”. https://www.merriam-webster.com/dictionary/lacuna.

## References

[B1] Bersagel A. (2012). Is there a stare decisis doctrine in the Courtof Arbitration for Sport? An analysis of published awards for anti-doping disputes in track and field. Pepperdine Dispute Law Resol. J. 12, 189–213.

[B2] Bock B. (2022). Bock: Why Kamila Valieva (A Russian Athlete Who Consumed a Serious Doping Substance) Should Be Allowed to Compete in the Olympic Figure Skating Competition on Tuesday. Available online at: https://kgrlaw.com/ (accessed May 12)

[B3] Broekman J. M. (1985). Beyond Legal gaps. Law Philos. 4, 217–235. 10.1007/BF00157089

[B4] Costa J.-P. (2019). Legal Opinion 2019 (Expert Opinion) on the World Anti-Doping Code. Available online at: https://www.wada-ama.org/sites/default/files/resources/files/avis_2019_code_mondial_en.pdf (accessed May 12)

[B5] Court of Arbitration for Sport Award. Mariano Puerta v. International Tennis Federation, CAS 2006/A/1025. Available online at: https://jurisprudence.tas-cas.org/Shared%20Documents/1025.pdf (accessed July 12 2005).

[B6] Court of Arbitration for Sport. Valencia Club de Fútbol S.A.D. v. Fenerbahçe Spor Kulübü, CAS 2018/A/5950. Available online at: https://jurisprudence.tas-cas.org/Shared%20Documents/5950.pdf.

[B7] Court of Arbitration for Sport. Anna Chicherova v. CIO, CAS/2016/4839. https://jurisprudence.tas-cas.org/Shared%20Documents/4839.pdf (accessed October 6 2017).

[B8] Court of Arbitration for Sport. Canas v. ATP Tour, CAS 2005/A/951. Available online at: https://jurisprudence.tas-cas.org/Shared%20Documents/951.pdf (accessed May 23rd, 2007).

[B9] Court of Arbitration for Sport. Ihab Abdelrahman c. ONAD d'Egypte, CAS OG 16/23. Available online at: https://jurisprudence.tas-cas.org/Shared%20Documents/OG%2016-023.pdf (accessed August 16, 2022).

[B10] Court of Arbitration for Sport. IOC, WADA, and ISU v. RUSADA, Kamila Valieva and Russian Olympic Committee, CAS OG 22/08, CAS OG22-09, CAS OG 22-10. Available online at: https://www.tas-cas.org/fileadmin/user_upload/OG_22_08-09-10_Arbitral_Award__publication_.pdf (accessed February 14, 2022) 10.3389/fspor.2022.946608 PMC9294144 35865485

[B11] Court of Arbitration for Sport. Justin Gatlin c. USADA, CAS 2008/A/1461. Available online at: https://jurisprudence.tas-cas.org/Shared%20Documents/1461,%201462.pdf (accessed July 12, 2005).

[B12] Court of Arbitration for Sport. Juventus Football Club S.p.A. v. Chelsea Football Club Ltd, CAS/2013/A/3365 *et A.S. Livorno Calcio v. Chelsea Football Club Ltd*, CAS/2013/A/3366. Available online at: https://jurisprudence.tas-cas.org/Shared%20Documents/3365,%203366.pdf (accessed January 21, 2015).

[B13] Court of Arbitration for Sport. Knauss v. FIS, CAS/2005/A/847. Available online at: https://jurisprudence.tas-cas.org/Shared%20Documents/847.pdf (accessed July 20, 2005).

[B14] Court of Arbitration for Sport. R. c. UEFA, TAS 2005/A/958. Available online at: http://www.centrostudisport.it/PDF/TAS_CAS_ARCHIVIO/191.pdf (accessed November 9, 2005).

[B15] Court of Arbitration for Sport. Advisory opinion of August, 31st 1994, Fédération Française de Triathlon (FFTri) & International Triathlon Union (ITU), TAS 93/109. Available online at: https://jurisprudence.tas-cas.org/Shared%20Documents/109.pdf (accessed May 12)

[B16] Court of Arbitration for Sport. Advisory Opinion of August, 31st 1994. AEK Athènes & SK Slavia de Prague v. UEFA, CAS 98/200. Available online at: https://jurisprudence.tas-cas.org/Shared%20Documents/200.pdf (accessed August 20, 1999).

[B17] Foriers P. (1967). Les lacunes du droit. Logique Anal Nouvelle Sér. 10, 37.

[B18] International Olympic Committee (2022). IOC EB Decides No Medal Ceremonies Following CAS Decision on the Case of ROC Skater. Available online at: https://olympics.com/ioc/news/ioc-eb-decides-no-medal-ceremonies-following-cas-decision-on-the-case-of-roc-skater (accessed February 14, 2022).

[B19] International Standard—Laboratories (2021). Available online at: https://www.wada-ama.org/sites/default/files/resources/files/isl_2021.pdf (accessed May 12)

[B20] Irti C. (2014). A short introduction to the problem of legal gaps. Tulane European and Civil Law Forum 29, 157–179. Available online at: https://journals.tulane.edu/teclf/article/view/1683 (accessed May 12)

[B21] Kauffman-Kohler G. (2007). Arbitral Precedent: Dream, Necessity or Excuse? Int. Arbitrat. 23, 357–378. 10.1093/arbitration/23.3.357

[B22] Lewis A. Taylor J. (2021). Sport: Law and Practice. London: Bloomsbury.

[B23] Lindolhm J. (2021). A legit supreme court of world sport? The CAS(e) for reform. Int. Sport Law J. 21, 1–5. 10.1007/s40318-021-00184-0

[B24] Maisonneuve M. (2017). Le Tribunal arbitral du sport et les droits fondamentaux des athlètes. *RDLF*, 2017 chron. n°09. Available online at: http://www.revuedlf.com/droit-international/le-tribunal-arbitral-du-sport-et-les-droits-fondamentaux-des-athletes/#note-6479-26 (accessed May 12)

[B25] Pavot D. (2021). La lacune en était-elle vraiment une? L'interprétationincertaine du Tribunal arbitral du sport dans l'affaire *Kamila Valieva* (CAS OG 22/08, CAS OG22-09 et CAS OG 22-10). Cahiers de Droit du Sport 61, 6.

[B26] Siorat L. (1959). Le problème des lacunes en droit international (contribution à l'étude des sources du droit et de la fonction judiciaire. Paris: LGDJ.

[B27] Swiss Federal Tribunal (2003). Lazutina v. CIO, 4P.267–270/2002.

[B28] Swiss Federal Tribunal (2014). A. v. B., 4A_90/2014. Available online at: https://www.bger.ch/ext/eurospider/live/fr/php/aza/http/index.php?highlight_docid=aza%3A%2F%2F09-07-2014-4A_90-2014&lang=fr&type=show_document&zoom=YES& (accessed May 12)

[B29] Troper M. (2011). Droit et nécessité. Paris: PUF.

[B30] World Anti-Doping Agency (2020). Report of the Independent Observers—Olympic Games Tokyo 2020. Available online at: https://www.wada-ama.org/sites/default/files/2022-01/IO%20Report_2020%20Tokyo%20Olympic%20Games_Final.pdf (accessed May 12)

[B31] World Anti-Doping Agency (2022). WADA Statement Following CAS Decision Not to Reinstate Skater's Provisional Suspension. Available online at: https://www.wada-ama.org/en/news/wada-statement-following-cas-decision-not-reinstate-skaters-provisional-suspension (accessed February 14, 2022).

[B32] World Anti-Doping Code 202 (2021). Available online at: https://www.wada-ama.org/en/resources/world-anti-doping-program/world-anti-doping-code

